# Associations Between Cognitive Performance, Depression, Anxiety, and Stress in Patients With Chronic Vestibular Dysfunction: A Cross-Sectional Analysis

**DOI:** 10.7759/cureus.95471

**Published:** 2025-10-26

**Authors:** Priscila Stefania Contreras Gonzalez, Victoria Sosa Romo, Daniel Ramos Maldonado, Ivonne Calderón Leyva, Annel Gómez Coello

**Affiliations:** 1 Department of Otoneurology, Sub-directorate of Audiology, Phoniatrics, and Language Pathology, National Institute of Rehabilitation Luis Guillermo Ibarra Ibarra, Ciudad de Mexico, MEX; 2 Department of Otolaryngology, National Institute of Rehabilitation Luis Guillermo Ibarra Ibarra, Ciudad de México, MEX

**Keywords:** cognition function, cognitive impairment and dementia, c-vemp, montreal cognitive assessment (moca), otoneurology, o-vemp, vestibular evaluations, vestibular evoked myogenic potentials, vestibular system

## Abstract

Background: The vestibular system is critical for sensorimotor function, balance control, and gaze stability. Recent research highlights its role in cognitive functions, including attention, memory, and visuospatial skills. The integration of this system is lateralized in the brain. These disorders represent a substantial burden on healthcare systems, and a negative impact on quality of life.

Objectives: Previous research has shown that increased cognitive load can alter vestibular evoked myogenic potentials (VEMPs), suggesting that vestibular function is influenced by cognitive demands. Therefore, this study aims to (1) examine the relationship between cognitive performance, as measured by the Montreal Cognitive Assessment (MoCA), and baseline vestibular function through cervical and ocular VEMPs (cVEMPs and oVEMPs), to determine whether vestibular dysfunction is associated with cognitive decline; (2) assess the association between depression, anxiety, and stress measured by the Depression, Anxiety, and Stress Scale (DASS-21) subscales, and VEMP parameters; and (3) explore the overall interaction between cognitive and affective domains in chronic vestibular dysfunction, providing insight into the neurophysiological links between balance, cognition, and emotion.

Methodology: Patient data was retrieved from the Otoneurology Service database at the National Institute of Rehabilitation Luis Guillermo Ibarra Ibarra, covering the period from January 2017 to December 2019. This study was conducted from 2022 to 2025. Data included: self-reported scores from the DASS-21 and results from the MoCA test. For data analysis, descriptive statistics, bivariate frequency tables, and the Shapiro-Wilks test, were used to characterize the variables. Spearman's correlation coefficients were then used to evaluate the relationship between MoCA scores, the scores from the DASS-21 scales (depression, anxiety, and stress), cVEMP, and oVEMP values, using a *P* value < 0.05 for statistical significance. Linear regression was subsequently performed to further analyze any relationships found to be statistically significant.

Results: In this study of 29 subjects, a demographic analysis revealed a slight female predominance, with 18 subjects (62.1%) female and 11 subjects (37.9%) male. The mean age was 49 years. The most prevalent diagnosis was right vestibular dysfunction, which affected 17 subjects (58.6%), with dizziness being the most common accompanying symptom, also reported by 17 subjects (58.6%). A subsequent Spearman correlation analysis revealed a positive and statistically significant (*P=0.04*), though medium, correlation between cognitive function (as measured by the MoCA) and the amplitude of the right ocular VEMP. This finding was further supported by a linear regression analysis, which also showed a statistically significant relationship between the variables (*P=0.03*).

Conclusions: A positive and statistically significant medium correlation was found, using both Spearman's correlation coefficient and linear regression, between cognitive function and amplitude of vestibular response in the right oVEMP. Conversely, this study found no significant correlation between vestibular evoked myogenic potentials, cervical or ocular, and the variables for the DASS-21. Though further research needs to be done to definitively determine the relationship between the vestibular system, cognition, and cognitive states, this study provides valuable preliminary insights.

## Introduction

The execution of most daily activities requires the integration of motor and cognitive functions, a process critically dependent on the vestibular system for maintaining postural stability. This functional equilibrium enables the central nervous system to interpret and synthesize sensory input from the environment. Beyond its traditional role in balance, accumulating evidence highlights the contribution of vestibular afferents to higher-order cognitive processes, including attention, memory, navigation, and visuospatial abilities [1-5]. Supporting this link, increased cognitive load has been shown to modulate ocular vestibular evoked myogenic potentials (oVEMPs), suggesting direct pathways between vestibular and cognitive networks [6]. Visuospatial cognition, in particular, is predominantly mediated by the right hemisphere through distributed networks spanning parietal, temporal, and frontal regions [7]. The right inferior and superior parietal lobules constitute key hubs for spatial attention, mental rotation, and navigation, with lesions often producing hemispatial neglect and contralateral (typically left-sided) perceptual deficits. Disruption of these networks can result in deficits in route finding, spatial memory, and environmental recognition [8]. When compromised, particularly in cases of peripheral vestibular dysfunction, the vestibular system's altered oculomotor and postural responses can lead to a vestibular syndrome. This syndrome is characterized by symptoms such as vertigo, nystagmus, ataxia, and postural instability [9]. The widespread nature of the cortical vestibular network means that such dysfunction can have generalized effects on neurocognitive function, regardless of whether the onset is acute or chronic. The clinical importance of vestibular disorders is highlighted by their high prevalence and significant economic impact. Recent epidemiological studies show that these disorders affect over 35% of adults aged 40 and older, with prevalence increasing to nearly 50% between the ages of 60 and 69, and to 69% in those aged 70 to 79. The economic impact is also considerable: a 2011 study estimated that 25.7% of all emergency department visits for dizziness or vertigo in the U.S. were attributed to otologic or vestibular causes, at an estimated cost of $757 million [10]. 

The effects of vestibular dysfunction extend beyond motor control, impacting a wide range of cognitive and emotional functions. Visuospatial ability, including skills such as spatial memory and navigation, are domains often compromised [3]. Vestibular loss also appears to impact attention and executive functions, which are foundational for planning and decision-making [11,12]. The interconnectedness of these systems is further suggested by a potential link between vestibular dysfunction and emotional disorders, as serotonin-releasing neurons project to both the amygdala and the brainstem's vestibular nucleus, influencing emotional state [9,13]. To assess these multifaceted effects, a comprehensive battery of tools is necessary. Vestibular evoked myogenic potentials (VEMPs), including cervical VEMPs (cVEMPs) and oVEMPs, are key for assessing vestibular function; they measure action potentials from the otolith system and electromyographic activity [14,15]. The process of assessing cVEMPs involves stimulating each ear separately to activate the vestibular organs. The electrical response is then measured from the ipsilateral sternocleidomastoid muscle (SCM) due to its accessibility. This response is a biphasic wave with two key components: the first positive peak at approximately 13 ms (P13) and the second negative peak at 23 ms (N23). The P13 wave represents the initial excitatory response, while the N23 wave reflects the subsequent inhibitory component. The difference between these two waves is described as amplitude. The oVEMP measures electrical activity in the extraocular muscles and is associated with the vestibulo-ocular reflex. Similar to the cVEMP, the oVEMP is characterized by two peaks: an initial negative peak n10 and a subsequent positive peak p15 [16,17]. 

Meanwhile, the Montreal Cognitive Assessment (MoCA) is a validated and comprehensive screening tool used to evaluate cognitive domains [18]. The MoCA assesses short-term memory, visuospatial abilities, executive functions, attention, language, and orientation, with a total score ranging from 0 to 30. A higher score reflects better cognitive function. A score of 26 or less was used as the cutoff for determining cognitive impairment [19]. Given that coexisting emotional states like depression, anxiety, and stress can influence cognitive test results, the Depression, Anxiety, and Stress Scales (DASS-21) has been used to account for the patient’s self-perceived emotional state [20]. The self-assessment consists of three scales with seven items each, with four alternative responses arranged on a scale from 0 to 3 points. The score for each scale is calculated by summing the scores of the items belonging to that scale. The degree of severity is determined based on the raw scores. 

A significant gap exists in the current body of literature concerning the intricate interplay between vestibular dysfunction, cognitive function, and psychological conditions. To bridge this gap, this study aims to examine the correlational relationship between vestibular function, as measured by VEMPs, using MoCA for cognitive function and DASS-21 for depression, anxiety, and stress.

## Materials and methods

Procedural description

Patient data were retrieved from the Otoneurology Service database at the National Institute of Rehabilitation Luis Guillermo Ibarra Ibarra (INR GII), utilizing records from January 2017 to December 2019. This initial screening identified patients with baseline cervical and ocular VEMP values. A subsequent follow-up period from 2022 to 2025 was established for patient recruitment. Patients were contacted by phone and invited to participate if their most recent vertigo recurrence had occurred within the previous three months. Upon verbal agreement, informed consent and assent were read aloud and subsequently signed. During an in-office visit, a researcher completed a data collection sheet with personal details and medical history.

The cVEMPs and oVEMPs were recorded individually. The cVEMP is an action potential from the saccule, which produces an inhibitory cervical potential recorded on the ipsilateral SCM. Each ear was stimulated separately with an acoustic stimulus delivered via air. The stimulus parameter was a toneburst at a low frequency (500 Hz) and high intensity (90-100 dB). The cVEMP appears as a biphasic wave with a positive P1 peak (at 13 ms) followed by a negative N1 peak (at 23 ms). The amplitude of this potential is directly proportional to the muscular contraction of the SCM, which is required to be at a minimum of 50 microvolts for the potential to appear [16]. While the stimulus is unilateral, the response is bilateral, consisting of an ipsilateral inhibitory potential and a small contralateral excitatory potential. The oVEMP represents the electromyographic activity of the extraocular muscles, associated with the vestibulo-ocular reflex. The stimulus is delivered via air or bone conduction. For air conduction, we used the frequency range of 400-800 Hz, while for bone conduction we used 100 Hz. Electrodes were placed near the lower eyelid to record the activity of the inferior oblique muscle. The patient was asked to look upwards, which increased the tonic activity of the muscle and consequently the amplitude of the potential. The potential consists of a series of waves with an initial negative peak (N1) and a second positive peak (P1) [15,17]. 

For the cognitive assessment, the MoCA, which was published and validated in 2005 by Nasreddine et al., was employed with permission [19]. The MoCA provides a total score ranging from 0 to 30, with higher scores indicating better cognitive function. A score of 26 or less was used as the cutoff for determining cognitive impairment; a score of 18-25 determined slight cognitive impairment; a score of 10-17 determined moderate impairment; a score of <10 determined severe cognitive impairment. On the day the MoCA was scheduled, the patient was given instructions for each item, which are as follows: a short-term memory task (5 points) consisting of two learning trials of five nouns and a delayed recall after approximately five minutes. Visuospatial abilities were evaluated with a clock-drawing task (3 points) and copying a three-dimensional cube (1 point). Multiple aspects of executive functions were assessed with a trail-making task (1 point), a phonemic fluency task (1 point), and a two-item verbal abstraction task (2 points). Attention, concentration, and working memory were evaluated with a sustained attention task (target detection with taps; 1 point), a serial subtraction task (3 points), and forward and backward digits (1 point each). Language was evaluated with a three-item confrontation naming task using less common animals (lion, camel, rhinoceros; 3 points), repeating two complex syntactic sentences (2 points), and the aforementioned fluency task. Finally, temporal and spatial orientation were evaluated (6 points) [19]. Patients were briefed on the basic materials (paper, pencil, eraser) to complete the test, and they could be assisted by a family member or caregiver.

Participants then completed the DASS-21. The self-assessment consists of three scales with seven items each, with four alternative responses arranged on a scale from 0 to 3 points. The score for each scale is calculated by summing the scores of the items belonging to that scale. The degree of severity is determined based on the raw scores as follows: Normal: Depression 0-9, Anxiety 0-7, Stress 0-14. Mild: Depression 10-13, Anxiety 8-9, Stress 15-18. Moderate: Depression 14-20, Anxiety 10-14, Stress 19-25. Severe: Depression 21-27, Anxiety 15-19, Stress 26-33. Extremely Severe: Depression >28, Anxiety >20, Stress >34 [20].

The sample size was determined using the Fisher Z-transformation formula, which is suitable for detecting correlations between two quantitative variables. This method converts the expected correlation coefficient into a normally distributed Fisher Z value, enabling accurate estimation of the number of participants required to achieve the desired confidence level and statistical power. In the absence of prior data, Cohen’s benchmarks were applied to estimate the effect size. A large effect (r = 0.50) was selected based on theoretical reasoning, reflecting a strong and biologically plausible association. To preserve adequate study power, the final sample size was increased by 10% to account for potential participant loss.

n= [(Zα/2+Zβ​)^2/(½ In [1+r/1-r])^2]+3

Significance level: α=0.05 = Zα/2=1.96; Power: 80% = Zβ=0.84; Expected correlation coefficient: r=0.50; Fisher z: 1/2In [(1+0.5)/(1-0.5)] = 0.5493; Sample size calculation: (7.84/0.3017)+3≈25+3≈28; Adjustment for potential 10% dropouts: final= 28x1.10≈31.

Statistical analysis

After the data were gathered, we began our analysis in Python version 3.12. First, we used the Shapiro-Wilk test to determine if the data were normally distributed. We then constructed bivariate frequency tables to explore the relationships between several key variables and to identify potential confounders for later removal. These tables specifically examined whether a subject's age affected their myogenic evoked potentials (VEMPs) and MoCA scores. In addition, we looked at the relationship between laterality and VEMPs, and between comorbidities and VEMPs, MoCA, and DASS-21 scores. Following this, we calculated Spearman's correlation coefficients to quantify the relationships between individual right and left VEMP scores (cVEMP and oVEMP) and the scores for MoCA and each subscale of the DASS-21 (depression, anxiety, and stress). Finally, linear regression was applied to any statistically significant relationships found during the correlation analysis. Using the threshold for p < 0.05 for statistical significance for both Spearman's correlation coefficients and linear regression. 

## Results

Twenty-nine subjects who met the inclusion criteria were included in the study. The sample was composed of 18 females (62.1%) and 11 males (37.9%), with an average age of 49 years and a standard deviation of 7.02. Regarding prior diagnoses, right vestibular dysfunction was the most common, affecting 58.6% of the sample, followed by left vestibular dysfunction at 24.1%, and bilateral vestibular dysfunction in the remaining 17.3% (n=5). A preliminary analysis using the Shapiro-Wilk test indicated that the variables for age, anxiety, and stress showed a normal distribution, while all other variables demonstrated a non-normal distribution (Table 1).

**Table 1:** Quartiles, interquartile range, median, and Shapiro-Wilk test results μV: microvolts; W: Shapiro-Wilk; Q1: quartile 1; Q2: quartile 2; Q3: quartile 3, DASS-21: Depression, Anxiety, and Stress Scale-21; MoCA: Montreal Cognitive Assessment; oVEMP: ocular vestibular evoked myogenic potential; cVEMP: cervical vestibular evoked myogenic potential Sex: feminine = 1, masculine = 0 Comorbidities: none = 0, diabetes or/and hypertension = 1 Laterality: right = 0, left = 1, bilateral = 2 Current symptoms: yes = 1, no = 0

	Sex	Age	Comorbidities	Laterality	Current Symptoms	right- CVEMP μV	left-CVEMP μV	right-OVEMP μV	left-OVEMP μV	MoCA score	Depression (DASS-21)	Anxiety (DASS-21)	Stress (DASS-21)
Shapiro-Wilk	W= .61, p < .001	W= .95, p = .222	W= .46, p < .001	W= .71, p < .001	W= .71, p < .001	W= .84, p < .001	W= .85, p < .001	W= .9, p = .010	W= .73, p < .001	W= .89, p = .006	W= .89, p = .006	W= .94, p = .086	W= .93, p = .051
Median	0	49	1	0	0	27.02	40.35	2.83	2.53	26	8	10	12
Interquartile range	1	11.5	0	1	1	22.33	74.27	5.05	5.88	1	10	12	11
Q1	0	43	1	0	0	17.77	17.11	0	1.05	26	3	4	9
Q2	0	49	1	0	0	27.02	40.35	2.83	2.53	26	8	10	12
Q3	1	54.5	1	1	1	40.1	91.38	5.05	6.93	27	13	16	20

A preliminary bivariate analysis revealed an association between only the right ocular VEMP and MoCA scores. Since no significant relationships were found with age, laterality, or comorbidities, these variables were not considered potential confounders for the relationship. We then calculated Spearman's correlation coefficients to quantify the relationships between right and left VEMP amplitude scores and the scores for DASS-21 with the variable of depression in Table 2, stress in Table 3, and anxiety in Table 4. 

**Table 2:** Spearman correlation coefficient DASS-21 (Depression) vs amplitude of cVEMPS and oVEMPS DASS-21: Depression, Anxiety, and Stress Scale-21; cVEMP: cervical vestibular evoked myogenic potential; oVEMP: ocular vestibular evoked myogenic potential; rS: Spearman's correlation coefficient; p: probability value (0.05)

DEPRESSION	cVEMP right	cVEMP left	oVEMP right	oVEMP left
rS	-0.14	-0.22	0.17	-0.23
p	0.47	0.25	0.56	0.22

**Table 3:** Spearman correlation coefficient DASS-21 (Stress) vs amplitude of c-VEMPS and oVEMPS DASS-21: Depression, Anxiety, and Stress Scale-21; cVEMP: cervical vestibular evoked myogenic potential; oVEMP: ocular vestibular evoked myogenic potential; rS: Spearman's correlation coefficient; p: probability value (0.05)

STRESS	cVEMP right	cVEMP left	oVEMP right	oVEMP left
rS	-0.00	-0.03	0.08	-0.19
p	0.99	0.89	0.66	0.33

**Table 4:** Spearman correlation coefficient DASS-21 (Anxiety) vs amplitude of cVEMPS and oVEMPS DASS-21: Depression, Anxiety, and Stress Scale-21; cVEMP: cervical vestibular evoked myogenic potential; oVEMP: ocular vestibular evoked myogenic potential; rS: Spearman's correlation coefficient; p: probability value (0.05)

ANXIETY	cVEMP right	cVEMP left	oVEMP right	oVEMP left
rS	0.08	-0.16	-0.04	-0.31
p	0.67	0.42	0.86	0.11

The findings from the Spearman correlation coefficient revealed no statistically significant correlation between scores on the DASS-21 and VEMP data. This suggests that depression, anxiety, and stress may not directly influence vestibular myogenic potentials. However, given the limitations of the current sample size, a larger study is needed to definitively confirm this lack of a relationship. 

Using the Spearman correlation coefficient (Table 5), a positive and statistically significant correlation was found between cognitive function, as measured by the MoCA, and the amplitude of the right oVEMP (*r=0.38756, P=0.0378*). This finding indicates that higher MoCA scores are associated with a more robust vestibular response in the right oVEMP.

**Table 5:** Spearman correlation coefficient MoCA vs amplitude of cVEMPS and oVEMPS MoCA: Montreal Cognitive Assessment; cVEMP: cervical vestibular evoked myogenic potential; oVEMP: ocular vestibular evoked myogenic potential; rS: Spearman's correlation coefficient; p: probability value (0.05)

MoCA	cVEMP right	cVEMP left	oVEMP right	oVEMP left
rS	0.00	0.12	0.39	-0.24
p	0.97	0.52	0.04	0.22

Finally, we applied linear regression to the variables of right ocular VEMP amplitude and MoCA scores (Table 6). This analysis confirmed the relationship found by the Spearman's correlation coefficient, revealing a statistically significant association (P=0.03). The regression model showed that 0.16 of the variance in MoCA scores can be explained by the amplitude of the right ocular VEMP. 

**Table 6:** Linear regression for oVEMP right and MoCA scores MoCA: Montreal Cognitive Assessment; oVEMP: ocular vestibular evoked myogenic potential; Sy.x: Standard Error of the Estimate; DFn: Numerator Degrees of Freedom; DFd: Denominator Degrees of Freedom

Best fit values	
Slope	0.49± 0.22
Y-intercept	-9.69± 5.7
X-intercept	19.8
1/Slope	2.04
95% Confidence Intervals	
Slope	0.04 to 0.94
Y-intercept	-21.47 to 2.11
X-intercept	-52.51 to 23.15
Goodness of Fit	
R Square	0.16
Sy.x	2.5
Significance	
F	4.98
DFn,DFd	1,27
P Value	0.03
Deviation from zero	Significant

**Figure 1:**
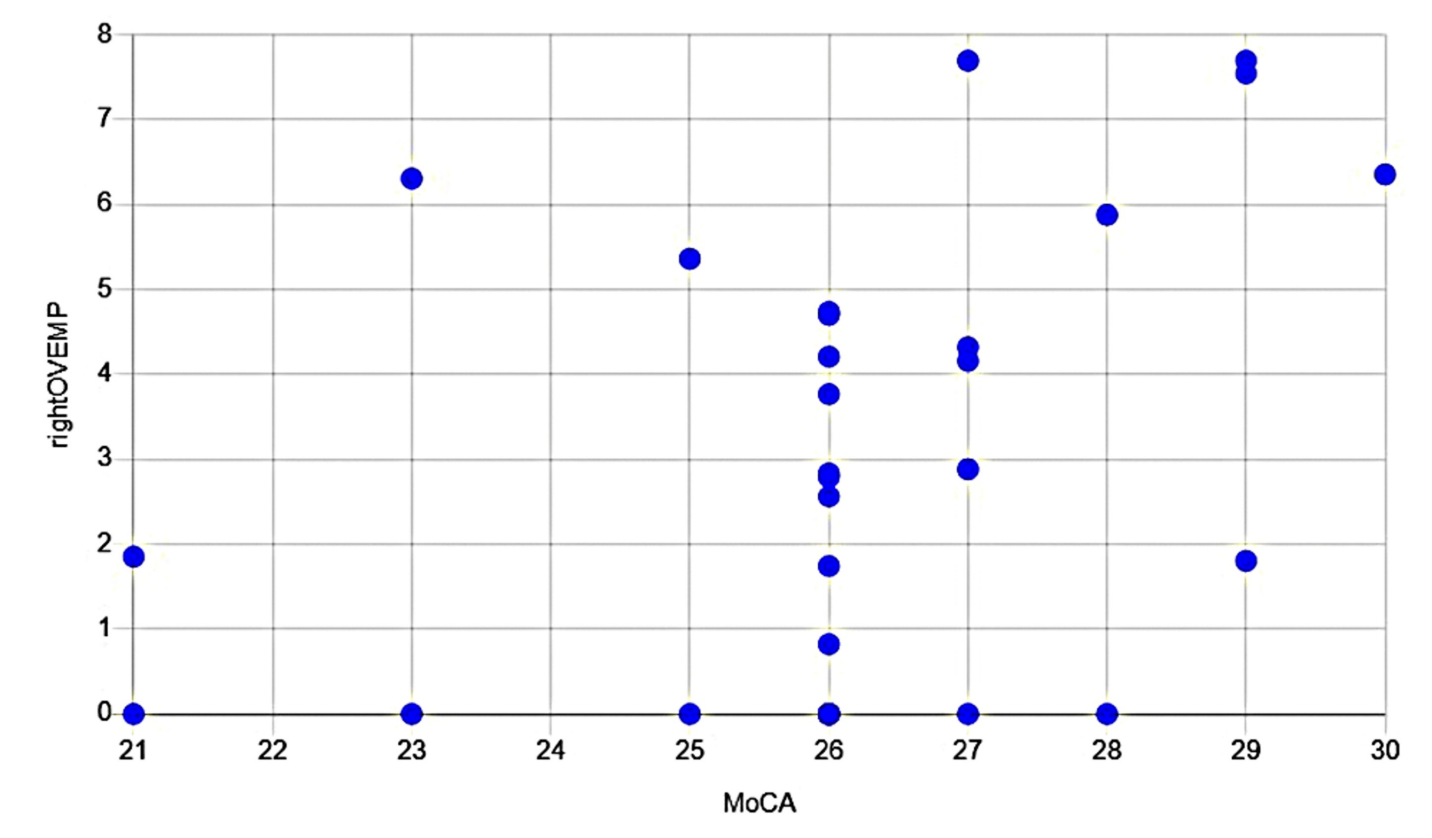
Spearman´s correlation coefficient for MoCA and oVEMP right MoCA: Montreal Cognitive Assessment; oVEMP: ocular vestibular evoked myogenic potential Correlation Coeficient: 0.4; p Value: 0.04

**Figure 2:**
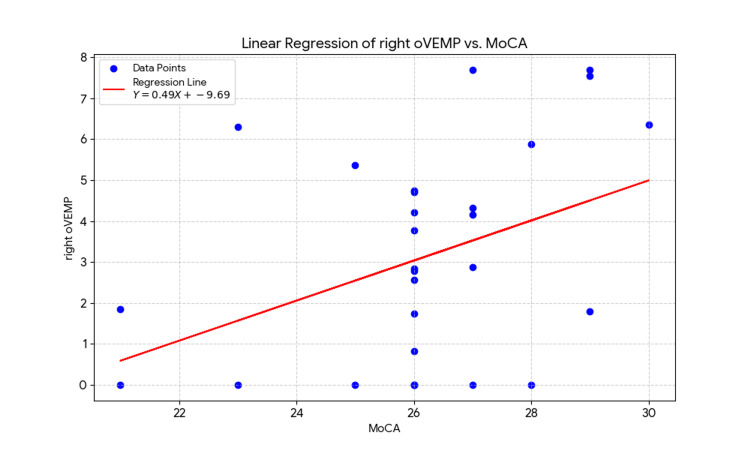
Linear regression for oVEMP and MoCA MoCA: Montreal Cognitive Assessment; oVEMP: ocular vestibular evoked myogenic potential Equation: Y = 0.4892*X - 9.685

These results suggest a meaningful connection between the vestibular system and cognitive function. The Spearman correlation finding (Figure 1) indicates that individuals with better cognitive performance, as measured by the MoCA, tend to have a more robust vestibular response in their right ocular VEMP. The linear regression (Figure 2) analysis further supports this relationship, showing that the amplitude of the right ocular VEMP accounts for 16% of the variation in MoCA scores (*P=0.03*). This suggests a significant link between the two systems, where the strength of the vestibular response plays a measurable role in cognitive performance.

## Discussion

This study aimed to investigate the relationship between vestibular function, cognitive health, and psychological states. Our findings show a positive and statistically significant correlation between cognitive function, measured by the MoCA, and the amplitude of the right oVEMP. This is supported by Spearman's correlation coefficient (rs​=0.4), as shown in Table 5 and Figure 1, and a linear regression analysis (R2=0.16) presented in Table 6 and Figure 2. This result suggests that a more robust vestibular response in the right oVEMP is associated with higher cognitive function, a finding that aligns with a growing body of evidence highlighting the critical role of the vestibular system in higher-order cognitive processes such as attention, memory, and spatial navigation [1,2,5,9]. This positive correlation reinforces the idea of a direct neural connection between the vestibular system and cognitive centers, as previously suggested by studies showing that heightened cognitive load can alter oVEMPs [6].

A particularly noteworthy finding is the lateralized nature of the correlation, which was significant for the right oVEMP but not the left. This provides support for the hypothesis that the right hemisphere plays a crucial role in visuospatial and other primary cognitive functions [18]. As the right hemisphere is predominantly responsible for visuospatial awareness, perception, and spatial representation, it is plausible that the functional relationship between the vestibular system and cognitive processing would be more pronounced on this side [7]. In contrast, our analysis revealed no statistically significant correlation between VEMP values and the Depression, Anxiety, or Stress subscales of the DASS-21, as detailed in Table 2 (Depression), Table 3 (Stress), and Table 4 (Anxiety). While the literature suggests a link between vestibular dysfunction and emotional disorders, our data did not support this association. This could be due to several factors, including the sample size or the specific population studied [13].

A major limitation of this study is the small sample size of 29 participants. This small number may have limited the power to detect weaker correlations and may not be fully representative of the general population of patients with vestibular dysfunction. Consequently, the findings should be interpreted with caution. Future research with a larger and more diverse cohort is needed to validate these findings and further explore the intricate relationship between vestibular function, cognitive health, and psychological well-being. The lateralized finding, where the correlation was significant for the right oVEMP but not the left, is particularly noteworthy. It provides support for the hypothesis that the non-dominant hemisphere - which for most individuals is the right hemisphere - plays a crucial role in visuospatial and other primary cognitive functions [18]. Since the right hemisphere is predominantly responsible for visuospatial awareness, perception, and spatial representation, it is plausible that a functional relationship between the vestibular system and cognitive processing would be more pronounced on this side. This lateralization of function may explain why the correlation was observed in the right oVEMP but not the left [7].

## Conclusions

This study found a positive and statistically significant correlation between right ocular VEMP amplitude and MoCA scores, a finding that was notably lateralized as no such relationship was found for the left VEMP. This result provides strong support for the hypothesis that the right hemisphere plays a crucial role in the integration of vestibular and cognitive functions, specifically those related to visuospatial and higher-order cognitive processing. The absence of a similar correlation with the left VEMP suggests a functional asymmetry in how the vestibular system contributes to cognitive health. While the small sample size is a limitation, this finding highlights the importance of considering laterality in evaluating vestibular function's contribution to cognitive health. The use of both Spearman’s correlation and linear regression strengthens the validity of this conclusion, providing complementary evidence that better cognitive performance is associated with a more robust vestibular response in the right ocular VEMP and providing a foundation for future research to validate these lateralized findings with larger cohorts.
